# The Risk of Eating Disorders in Adolescent Athletes: How We Might Address This Phenomenon?

**DOI:** 10.3390/sports12030077

**Published:** 2024-03-08

**Authors:** Isabel Cristina Rojas-Padilla, Iago Portela-Pino, María José Martínez-Patiño

**Affiliations:** 1 Faculty of Educational and Sports Sciences, National School of Sports, Cali 760042, Colombia; 2 Department of Special Didactics, Faculty of Education and Sports Sciences, University of Vigo, 36005 Pontevedra, Spain; mjpatino@uvigo.gal; 3 Department of Sport Sciences, Faculty of Health Sciences, Isabel I University, 09003 Burgos, Spain

**Keywords:** eating disorders, adolescents, athletes, risk factors, sport, prevention

## Abstract

Eating disorders are psychiatric and behavioral health pathologies of high complexity and different etiology, which can affect age groups, sexes, and ethnicities indistinctly. This study aimed to evaluate the risk of eating disorders and the possible relation with the sports profile of Colombian adolescent athletes. This was an exploratory cross-sectional quantitative study that used an online form designed with a sociodemographic questionnaire and the EAT-26 scale to determine the eating disorder risk of the object population. A total of 354 adolescent athletes participated. There were 182 men and 172 women and the mean age was 15.59 (range: 10–19 years, SD = 1.938). The participants presented a significantly low risk of eating disorders (21.2%) with no differences in prevalence between both sexes. The risk of eating disorder was related to the result of the last competition (*p* = 0.01), the type of sport (*p* = 0.032), the years of sports practice (*p* = 0.004), and the number of training hours a day (*p* = 0.011). It is relevant to recognize that adolescents and athletes are vulnerable populations regarding eating disorders. In conclusion, adolescent athletes should be the object of special attention to prevent eating disorders and their consequences on health and sports performance.

## 1. Introduction

Eating disorders (EDs) are abnormal attitudes and behaviors related to food intake [1]. These disorders share symptoms and mental origins as an excessive preoccupation with food, weight control, body image, and the use of different measures to control them [2]. To overcome this health condition, it is necessary to accept it, seek help from a professional clinical team, and have family support. Eating disorders generate irreversible sequelae [3], as they are unknown by many people and hidden by others [4].

The risk of suffering from an ED can occur at any stage of life; however, people between 10 and 19 years of age [5] are considered a highly vulnerable population since they face a period in which most of the changes in an individual occur, not only physical, but also emotional and social. According to the World Health Organization [6], one in every six people in the world is between 10 and 19 years of age; one in seven suffers from a mental disorder, generating 13% of the global burden of disease in this age group. Eating disorders occur in between 0.5% and 4% of adolescents worldwide [7], and they are more than twice as prevalent in females (3.8%) than males (1.5%) [8].

The presence of EDs in young people can affect different psychosocial and health spheres, generating social isolation, family conflicts, weak self-concept, low self-esteem, low autonomy, and fragility in intimacy, as well as alterations of the cerebral cortex [9,10]. In addition, it can compromise the different body systems affecting growth and development [11,12], which could influence young people to not reach their final height [13] or present decreased bone mineral density and brain atrophy [14]. Eating disorders can also alter organs and body parts, such as the skin [15], mouth [16,17], and pharynx [18]. Studies in Colombia have shown the prevalence of eating disorders in school adolescents with a difference regarding male and female sex [19] and also without any differences [20]. A high prevalence of ED was also found in university students [21,22]. In the international context, some studies have reported a higher prevalence of ED in female adolescents [23,24,25], while other research has reported no differences of ED between sexes [26,27].

In the athletic context, eating disorders are related to Relative Energy Deficiency in Sport (REDs), a common fact in female and male athletes representing various sports at different performance levels, causing problematic low energy availability (LEA) [28]. LEA induces the Female Athlete Triad with menstrual dysfunction, low energy availability, and low bone density, with some early symptoms such as anemia, depression, hypothermia, and excessive fatigue [29] in women who drastically decrease in weight. In men, LEA can originate in the Male Athlete Triad (MAT), represented by low energy availability, low bone mineral density, and low testosterone [30]. As a result, these triads impact potency, libido, and muscle strength [31]. Other consequences of ED are growth hormone resistance, impairing anabolic function for protein synthesis [32], decrease in muscle health [33], reduction in muscle protein, and depletion of body fluid levels and glycogen deposits [34], affecting the physical qualities of sports, such as endurance, power, and muscle strength [35]. Additional complications that athletes with ED may face are endocrine, urinary, and reproductive, as well as bone fragility due to bone demineralization, loss of skin oils, brittle nails, tooth decay, weak gums, slowed gastric and intestinal emptying with pain, constipation, gas, and low energy availability [36,37], which undoubtedly makes the athlete unfit for training and competition and contributes to deteriorating their health and athletic performance. Moreover, athletes with this condition would be exposed to cardiovascular problems and deterioration of the immune system [9], which could increase their vulnerability to getting sick and result in them missing out on training or competition, impacting their personal and family life, motivation, and anxiety, as well as generating an economic expense for the individual and their family.

Regarding this, a high risk of EDs has been found in athletes in general [38,39], and in specific sports such as swimming [40], combat sports [41], cheerleading [42], gymnastics, synchronized swimming, and ballet [43], sports with a body implication towards thinness and a weight-dependent discipline [44,45]. It is not only the type of sport that influences ED but also gender and although women may be more vulnerable [46,47], EDs occur in men due to their susceptibility to physical injury [48], situations that affect their training, and the competition process. Sports context pressures regarding weight and body appearance play an influential role in EDs [49].

The prevalence of EDs is important not only for the impact on public health but also for the repercussions in the context of adolescent sports, as these are the young people who are projected for high sporting achievements and generational renewal of athletes in the country. Therefore, the interest and importance of this study is justified by its contribution to the development of variables that should be considered in sports to avoid injuries, health problems, and poor athletic performance, thus increasing the possibility of obtaining high sporting achievements. Additionally, it highlights the need to know the behavior of athletes to implement actions to improve their lifestyles, as well as to contribute to the areas of science applied to sports in the fields of education, nutrition, and psychology, which contribute to obtaining high sporting achievements.

Consequently, the objective of this study was to evaluate the risk of eating disorders and the possible relationship with the sports profile (Last competition results, Level of competition, Sports sector, Type of sport, Hours of training, Number of trainings per day, Training days per week, and Years of sports practice), social profile (Institution, Level of institution, Socioeconomic stratum, and Media of transportation), and personal variables (Age, sex, housing area, and educational level) of Colombian adolescent athletes, since it is important to understand this population better in order to help in the prevention of EDs.

Therefore, the starting hypotheses aimed to confirm that there is an influence of the sports, social, and personal variables on the incidence of eating disorders in Colombian adolescents who practice competitive sports.

## 2. Materials and Methods

### 2.1. Study Design

A descriptive observational study with an exploratory nature was used, since the aim was to describe the presence, characteristics, and distribution of a phenomenon with a specific population. No type of intervention was carried out; we have limited ourselves to measuring the risk of ED in an intentional sample of adolescent athletes using a quantitative procedure and to locate the possible intervening variables in this process.

### 2.2. Participants

The sample consisted of 354 athletes: 182 men (51.4%), and 172 women (48.6%) between 10 and 19 years of age who practiced collective and individual sports disciplines. To participate in this study, the athletes must belong to a national league and be enrolled in the national diagnostic program, “Talentos Colombia”. Additionally, the participants must have been training at least for 2 years, with no injuries, surgery, or medical treatment during this period. Finally, the participants had to fill out the entire online form and attach the signature of the informed consent and assent form. These criteria were chosen because, in this study, we wanted to include a representative sample of athletes who represent the country in sporting events and those who are the national and international projection.

### 2.3. Study Questionnaire

To determine the athletes’ social, personal, and sports profile, we used questions about the athletes’ characteristics. As those questions were based on the questionary format of the “Talentos Colombia” program, it was possible to generate a report about the variables related to the risk of eating disorders and enrich the databases of the government program.

To determine the risk of eating disorders, the EAT-26 Scale (Eating Attitudes Test) was used, which was introduced in its short version by Garner (1982) [50]. It is the most widely used screening measure to help determine whether a person is at risk of suffering from an eating disorder and thus identifies whether they need professional attention. It is a measure of the symptoms and concern characteristics of eating disorders and is not used to make a diagnosis but is a particularly useful tool to assess the risk of suffering from one. It is a widely used and highly evaluated tool in young populations [51,52] and adolescent athletes [53,54,55,56]. The EAT-26 is self-administered and is answered using a Likert-type scale with 6 frequency categories: always, almost always, frequently, sometimes, rarely, and never. The first 25 questions have a favorable direction for the variable: the categories of never, rarely, and sometimes are scored with 0 points; frequently is scored with 1 point; almost always is scored with 2 points; and always is scored with 3 points. Question 26, which is unfavorable to the variable, is scored with 0 points for always, almost always, and frequently; 1 point for sometimes; 2 points for rarely; and 3 points for never. The total can vary between 0 and 78 points and a score of 20 or more indicates a risk of eating disorders [57]. This information allows us to group the ED variable into those who are at risk of presenting with ED (scores of 20 or higher) and those who are not at risk (scores less than 20).

For the implementation in Colombia, cultural, semantic, and factorial validation was considered in addition to the reliability assessment in the female population in this country, which presented a Cronbach’s alpha of 92.1%, a sensitivity of 100%, and a specificity of 85.6% [58]. On the other hand, for the Colombian male population, semantic, cultural, and factorial validation, reliability assessment, and determination of the best cut-off point using the ROC curve were performed in the population over 14 years of age. This validation found a Cronbach’s alpha of 0.89, a sensitivity of 100%, and a specificity of 97.8% with a predictive value of 91.3 and a negative value of 100% [59]. Therefore, these studies showed excellent reliability, sensitivity, and specificity values, which were ideal for screening for possible eating disorders and for use in primary care in both sexes. This survey has been used in several studies to determine the risk of eating behavior disorders in adolescents in the Colombian population, such as the study of Londoño and Moreno in 2017 in adolescents between 11 and 18 years of age, as well as in adolescent athletes in various areas of the world [40,41,43,60,61,62]. In this study, the scale reached a reliability value of 0.871 through Cronbach’s alpha to determine the non-risk risk of eating disorders.

The EAT-26 was chosen for this study because it has excellent reliability and sensitivity values, and adequate specificity values; this scale is appropriate for the objective of this study in a population who are vulnerable to EDs. Additionally, it is self-applicable, useful in primary care, and has been validated in Colombian adolescents and athletes.

### 2.4. Data Collection

The first step in the procedure was the design of an online form divided into three parts. The first part contained a detailed description of the research, highlighting the objective, the methodology, and the voluntary nature of participation, which the participants accepted by signing the informed consent and assent forms. The second part included the questions that would allow the socio-demographic characterization of the population, and the third part contained the EAT-26 survey.

Initially, a meeting was held with the methodologists and trainers to share the idea of conducting the research and to obtain their permission to carry it out. Afterwards, the form was sent to the athletes’ e-mail accounts along with a letter of invitation and the link to access the form.

Coaches assigned a specific space and day to perform the exercise. Fifteen minutes before starting their training, the athletes had a space to fill out the questionnaire. The idea was to be alone and concentrate on answering the questions. As everything was arranged, on that day, the athletes had to complete the assent and informed consent form, and a mobile device was available to access the questionnaire. The quality and accuracy of the responses were ensured by generating the context of the importance of the research, the confidentiality of the data, and the nature of voluntary participation.

This research was approved by the ethics committee of the National School of Sports in Colombia by number 17.133 on 11 May 2022. Participation was voluntary and accepted by signing an informed consent and assent form ensuring confidentiality through numerical codes and file encryption.

### 2.5. Statistical Analysis

Data analysis was carried out using the SPSS version 20 statistical package. In the univariate analysis, measures of central tendency (mean, standard deviation, minimum, and maximum) were calculated for the quantitative variables and frequencies, and percentages were used for the qualitative variables. For the bivariate analysis, the correlation of different variables was observed: risk of eating behavior disorder, years of sports practice, and the result of the last competition, which were calculated using Pearson’s correlation test, having verified that the assumption of normality was met through the Kolmogorov–Smirnov (KS) test. A student’s *t*-test was used to compare the risk of eating behavior disorder and to check possible statistically significant stocks when stratifying by sex. An ANOVA was used to determine the existence of statistically significant differences between the means of various groups; the three main variables: risk of eating behavior disorder with the variables educational level, sector of study, socioeconomic stratum, and means of transportation used by Colombian adolescent athletes. To establish an explanatory model of eating disorders in Colombian adolescent athletes as a function of sports and personal variables, given the characteristics of the variables, a multiple linear regression model was used, since several independent variables were expected to influence the dependent variable (ED).

Cohen’s d was also calculated to establish the effect size. Cohen’s criterion, which establishes three cut-off points for interpreting the effect size, were considered. In this sense, Cohen’s d values < 0.20 indicate no effect; values between 0.21 and 0.49 indicate a small effect; values between 0.50 and 0.70 indicate a moderate effect; and values > 0.80 indicate a great effect. Therefore, the departure of the TE from zero implies the rejection of the null hypothesis.

## 3. Results

### 3.1. Sociodemographics of Participants

The participants in this study were 354 athletes: 51.4% (n = 182) male and 48.6% (n = 172) female. The mean age was 15.59 years (maximum = 19; minimum = 10 (SD = 1.938)). The sampling was intentional to select a sample of Colombian adolescent athletes considering gender and different sports disciplines, both individual and collective.

The athletes who participated were part of sports leagues from different regions of the country, reporting a majority participation from the Pacific region, the western departments of the country (Valle, Chocó), and the Andean departments located in the central zone of Colombia (Cundinamarca, Antioquia, Boyacá), with 58.8% (n = 208) and 34.5% (n = 122), respectively. Regarding the housing area, 81.1% (n = 287) lived in an urban area and the predominant socioeconomic strata in the population were the three lowest (1, 2, and 3) with 79.9% (n = 318). The socioeconomic strata are classifications of residential properties in the cities of the country that are ranked from the lowest (stratum 1) to the highest (stratum 6). When asked with whom the athletes lived, it was found that most of them lived with their grandmothers and brothers, 51.1% (n = 181) and 37.3% (n = 132) respectively.

Considering schooling, 87.6% (n = 310) of the population was studying at the time of the survey; 64.4% (n = 228) of the participants were studying in public institutions and 23.2% (n = 82) were studying in private institutions. As for their level of schooling, 86.2% of the athletes (n = 305) were studying in secondary school, grades six to eleven, and only 0.3% (n = 1) were pursuing professional studies. The predominant means of transportation among the participants was walking with 39.8% (n = 141), the second was motorcycle with 22.6% (n = 80), and the least used was bicycle with 10.2% (n = 36) of the athletes.

In terms of health, we inquired about the athletes’ pathologies and those suffered by their first-degree relatives. Of those mentioned in the form, 5 were obese, 4 had a chronic disease (diabetes, arterial hypertension, or dyslipidemia), 3 had depression, 2 had pulmonary disease, 1 had epilepsy, and 1 had hydronephrosis. On the other hand, 76.9% (n = 272) of the athletes indicated having no family health history and among the 23.7% (n = 82) with a family health history, the most reported disease was cardiovascular disease in 8.2% (n = 29) and 6.5% (n = 23) had more than one chronic disease. 

It is possible to differentiate between kinds of sports: running sports (racing, skating, track and field, and para-athletics) with 32.4% (n = 115); ball sports (basketball, soccer, and volleyball) with 30.2% (n = 107); combat-level sports (boxing, judo, karate, wrestling, and taekwondo) with 13.8% (n = 49); strength sports (rugby and weightlifting) with 12.1% (n = 43); water sports (underwater, diving, conventional swimming, and para-swimming) with 3.2% (n = 11); art and movement sports (rhythmic and artistic gymnastics) with 2% (n = 7); racquet sports (badminton and tennis) with 0.8% (n = 3); table sports (chess) with 0.8% (n = 3); and bicycle sports (BMX cycling and road cycling) with 4.5% (n = 16).

It is important to mention that the sports sector with the highest response was the conventional sector with 92.7% (n = 328), although the invitation to participate in the study was extended to the Paralympic sector. Similarly, it was possible to classify the sports into two groups; it is evident that 67.8% (240) of the athletes practice an individual discipline. Additionally, 64.4% (n = 228) of the participants reported having been on the podium in their last competition, 91% (n = 322) of which were at the national level.

Concerning training time, we found that the years of practice was approximately 6 ($\bar{x}$ = 5.91; SD = 2.53) and they trained several days a week ($\bar{x}$ = 5.16; SD = 1.09), with at least one training per day ($\bar{x}$ = 1.29; SD = 0.58) and approximately 2 h for training per day ($\bar{x}$ = 2.25; SD = 0.57).

### 3.2. Eating Disorders

Considering the objective of this study: to identify the risk of eating disorders in Colombian adolescent athletes and using the EAT26, the prevalence of the risk of ED was found in 21.2% (n = 75) of the respondents (SD = 11.961; $\bar{x}$ = 13.09) (Table 1).

Of the 75 adolescents who were at risk of an eating disorder, 50.6% were male (n = 38) and 49.4% (n = 37) were female. As we can see, the difference is not significant.

When determining which variables influenced this prevalence, we found that there were no significant differences between the groups except with respect to the level of the last competition and the type of sport, which were higher in athletes who competed at the international level and in team sports (Table 2). If we focus on the effect size, we can see that the difference in the means of the significant variables was very strong (Level of competition; d = −7.52; Type of sport; d = −2.907).

When analyzing the variable risk of eating disorders with the personal variables (grouped age, educational level, sector of study, socioeconomic stratum, and means of transportation used by Colombian adolescent athletes), no significant differences were observed in any case (Table 3).

If the variable of type of sport with ED (risk, no risk) was considered, it was found that there was a significant association between both variables (X^2^ = 0.070; sig. = 0.040), as predicted using the calculation if it was used as the scale type variable.

When establishing the relationship between the risk of eating disorders with the sports profile (training, years of sports practice, hours of training, result of last competition) of the Colombian adolescents, a significant positive relationship was found between the variable and the hours of training (*p* = 0.001; r = 0.136), and the number of training sessions (*p* = 0.004; r = 0.153). No significant relationship was found with the variables of age, result of the last competition, or days of training per week (Table 4).

Therefore, we can affirm that the hypotheses of the influence of sports variables such as level of competition, type of sport, hours of training per day, number of trainings per day, and years in the sport on eating disorders in Colombian adolescent athletes are accepted. The hypotheses that predicted the influence of personal variables (age, sex, housing area, or educational level), sports profile (sports sector, last competition result, or training days a week), or social profile (institution, level of institution, socioeconomic stratum, or media of transportation) were not accepted.

**Figure 1:**
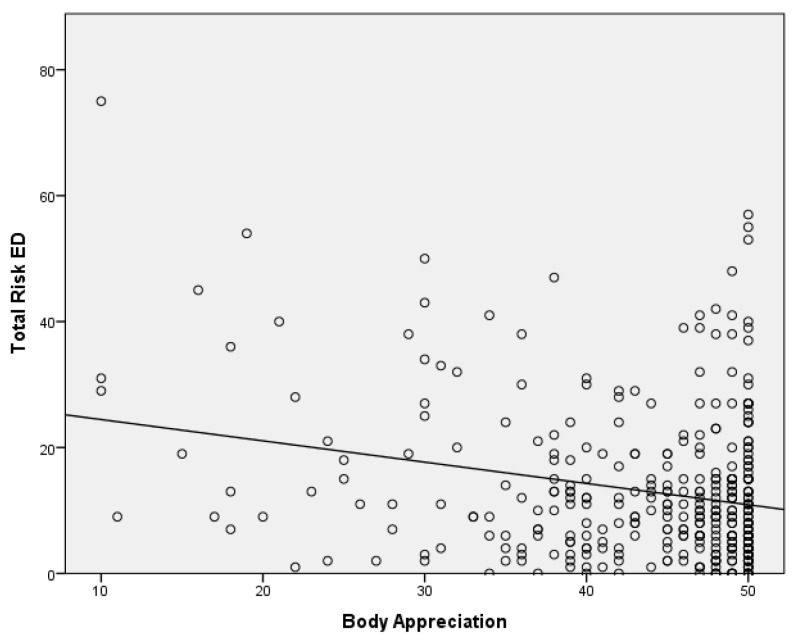
Risk of eating disorder based on years of sports practice in Colombian adolescent athletes participating in this study. N = 354, R^2^ = 0.057.

**Figure 2:**
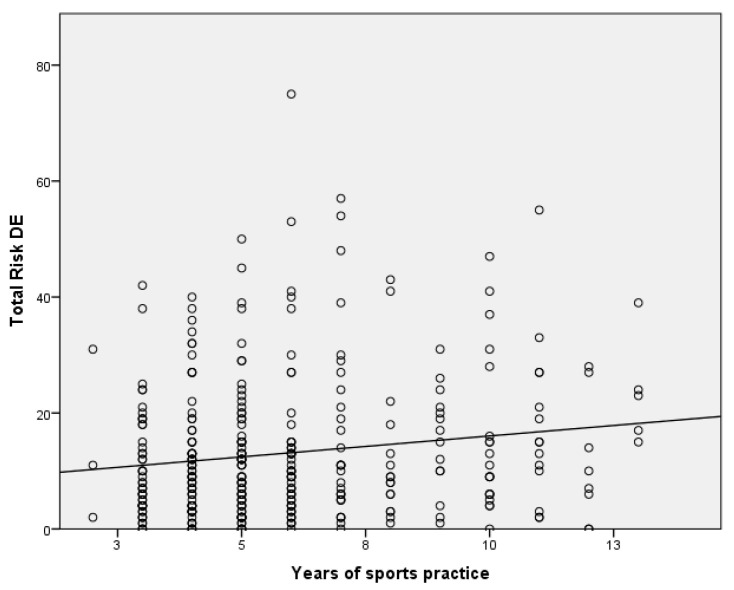
Risk of eating disorder based on body appreciation in Colombian adolescent athletes participating in this study. N = 354, R^2^ = 0.023.

Based on these results, an attempt (Table 5) has been made to establish an explanatory model of eating disorders in adolescent athletes. On the one hand, the expected sign of each variable regarding the dependent variable (DE) is included. Both the estimated (unstandardized) and the typed (standardized) coefficients of the model, called β, are included. The third and fourth columns present the values of the statistic and their significance (*p*-value < 0.05). The last column shows FIV values, which are less than 10; this suggests that there is no multicollinearity or internal correlation between the independent variables. Only the variables that were significant in the inference test performed have been included in the model (Figure 1 and Figure 2). Once the collinearity between the variables had been checked, only the variable “years of sport practice” was included, since there was collinearity with the number and hours of training per day. This disorder is explained using the variables of body appreciation (β = −0.245) and, to a lesser extent, years of sports practice (β = −0.163) and the type of last competition (i.e., national or international) (β = 0.164). The R^2^, which represents the percentage of the variation in the response variable that is explained using a linear model, reached 10% in this case.

If it is considered that the explanatory variable has significance in the model, the standardized regression coefficients for each direct route proposed in the model reached significance at the *p* > 0.05 level. Body appreciation had a negative slope, so if body appreciation increased, the risk of ED decreased (R^2^ = 0.057), sharing 5.7% of the variance; while the years of sports practice had a positive slope, so as the years increased, the risk of ED increased (R^2^ = 0.023). Both variables shared a variance of 2.3%. The independence of the errors has been proven as the Durbin–Watson value was 1.82.

Therefore, based on the results obtained, we can affirm that the eating disorders of Colombian adolescent athletes are associated with: (i) their body appreciation; (ii) the years of sports practice; (iii) and the scope of the competition in which they participate.

## 4. Discussion

### 4.1. Sociodemographics

The majority of Colombian adolescent athletes have a median age of 15.59. It is important to mention that a very high percentage of the athletes live in urban areas, which is related to the fact that the sports leagues (those organizations that govern the country’s sports clubs) develop their activities through the Inderes (Institutes of Sports and Recreation of the cities, especially the main ones), which are in the main cities. Additionally, transportation from rural areas to the training locations is usually difficult due to the distances and the state of the roads.

Similarly, most of the participants move on foot and another large percentage by motorcycle; these aspects that indicate that the purchasing power is not so high, an aspect that is observed in the socioeconomic status of the athletes, who are among the lowest. A different issue was found in a Brazilian study in which there was a high prevalence of socioeconomic high stratum in the athletes [63].

In terms of the people with whom the adolescents live, there is a predominance of living with grandparents and siblings. This may be related to an aspect mentioned in the previous paragraph, being from rural places but training in a sport, as due to the distance and transportation, athletes must move with relatives in the city. It may be also related to a common phenomenon in Colombia, where people migrate to other countries in search of better life opportunities. So, parents travel outside of the country to work and the children stay with their grandparents [64]. Living with grandparents is common in Colombia and this could be due to the socioeconomic status of the families or cultural aspects. Moreover, some studies have determined the importance of family coexistence in the incidence of eating disorders as eating patterns can be repeated or influenced by older adults in the family [65,66,67,68,69].

Regarding the health status of the athletes, a very high percentage reported not suffering from any disease. This could be expected because people who practice sports are usually healthy, since physical activity is a protective and disease-preventive factor [70]. Regarding their parents, the results indicate something similar. So, it could be suggested that there is no great weight in the family background.

On the other hand, concerning schooling, it is striking that 44 participants were not studying, when this population is at an age when they should generally be finishing the first cycle of primary school (first to fifth) and finishing secondary school (sixth to eleventh); a large percentage of the participants are even beginning their university studies. Most of the athletes studied in public institutions, which is closely related to their socioeconomic stratum discussed above.

These results showed what was expected in terms of training, which was several days a week due to the need for physical conditioning and maintenance, and improvement in technique, endurance, and discipline [71]. It was also found that, in some cases, there was practice more than once a day. This is because, in some sports, different kinds of training are performed, such as physical training in the gym and specific field training on the stage, or if they are in a pre-competitive cycle. Another result indicates that some of the participants have been practicing for up to 13 years, which shows that they have been training since they were in their early childhood.

### 4.2. Eating Disorders

This study found a prevalence of a 21.2% risk of ED. In other countries, this prevalence in adolescents is relatively low on average: Ecuador 15% [24]; Paraguay 9.5% [27]; and Spain 9.1% [72].

In adolescent athletes, suffering from EDs can lead to disease beyond the disorder itself and can lead to decreased muscle health, dehydration, low energy availability, and low glycogen resources [34]. In female adolescents, those health conditions could be combined in the athlete’s triad: low energy availability, bone weakness, and problems with their menstrual periods [29], due to their uncontrolled weight loss [33], and exposure to cardiovascular problems and immune system impairment [9]. In this study, the prevalence of the risk of ED in adolescent athletes was 21.2%. This percentage coincides with other studies in our country: 27.4% in karate; 50% in female boxers, and 52.9% in female wrestlers [41]. In other countries, the percentages are very different: Peru reported a 77.4% risk of ED in gymnastics, synchronized swimming, and ballet sports [43], and 34.4% was reported in cheerleaders from the United States [42]. To discuss these findings, it would be necessary to consider the cultural and sports differences.

If we consider the differentiation by sex, although years ago ED was characteristic of women [9], men are also suffering from this type of disorder [48]. In fact, in this study, 50.6% of men were at risk of ED; however, other studies [38] report a low prevalence in men (13.4%) and a higher prevalence in women. Although the percentage difference is significant, we must consider the differences in the characteristics of the populations under study. The case of adolescent athletes is unique because both sexes have similar hobbies, ambitions, and illusions, and in their eagerness to fulfill them they can follow recommendations that are not the most appropriate. However, when it comes to elite athletes like those reviewed in this research [38], it could be thought that they already have a greater knowledge of the consequences that could occur in health and performance if they suffer from an eating disorder. In men, a prevalence of 20.36% of ED risk was found in a study in Spain of athletes in which there was no difference between the team and individual disciplines of aesthetics and weight categories [64]. There was no difference in sports in which weight is relevant. For women athletes, this study revealed a 49.4% presence of ED in this sex. This was a much higher score than in Indonesia with 37.2% [44] and in London, with 16% in long-distance runners [39].

When correlating the risk of eating disorder with the sports profile (training hours per day, years of sports practice, and result of the last competition), there was a significant relationship regarding time of training per day and the years of sports practice. This issue may be related to the responsibility and awareness of athletes as they started training at a very young age. To win a championship, athletes should train with high intensity and frequency, generating a high energy expenditure [73]. On many occasions, the caloric requirement is not met due to schedules, eating strategies, quality, or quantity of food, which triggers an ED [74].

On the other hand, when comparing the risk of EDs with sex, area of residence, podium, type of last competition, sports sector, and type of discipline (individual sport and collective sport). Only the level of the last competition was statistically significant. It leads us to think that competing nationally or internationally may influence the result of the risk of ED. Studies have shown that the level of competition and training experience are related to the development of eating disorders in athletes [75,76]. Competing at the international level generates higher level standards in athletes, so this has been determined as a high-risk factor for presenting with eating disorder symptoms with the frequency [77,78,79] due to the sports and expectative requirements.

In this study, it was not found that there was any relation to the type of sport. Athletes such as artistic swimmers must face the evaluation of judges who emphasize body shape and performance in addition to technique and other evaluation criteria [60] that could address eating disorders. However, sports that involve aesthetics [75] and those that do not [80] can influence EDs; everything will depend on the personal and social context of the athlete.

Finally, when comparing the risk of EDs with the variables of educational level, sector of study, socioeconomic stratum, and means of transportation used by Colombian adolescent athletes, it was not found to be statistically significant. In the literature, it has been shown that in the high socioeconomic stratum, there is a greater probability of suffering this risk [9], which would lead to this being a protective factor for ED, since most of the population belongs to low socioeconomic strata.

People of low socioeconomic status may represent an elevated risk for EDs, given the associations between food insecurity and homelessness [81], and insufficient governmental benefits to meet monthly nutritional needs [82]. Barriers for people with low socioeconomic status include limited financial resources for healthcare and reduced access to mental health services for EDs [83]. Additionally, due to historical stereotypes that framed EDs as diseases of high socioeconomic status, people with lower socioeconomic status with ED symptoms are less likely to perceive a need for treatment [84].

## 5. Conclusions

This study contribution is novel since it determines the implication of variables such as hours of training per day, years that the participants have been practicing their sport, and the level of their last competition, in the possibility that an athlete suffers eating disorders.

The adolescent athletes participating in this study presented a significantly low percentage of eating disorders risk, with no differences in prevalence between both sexes.

The personal variables, such as socioeconomic stratum, housing area, study sector, educational level, and means of transportation did not show any influence on the risk of ED.

This study can provide ideas for continuing investigation into the personal and sports variables that could influence the adolescent’s journey through the sport, regarding the risk of eating disorders, as well as be taken into consideration as part of the pursuit of high sporting achievements and generational renewal of athletes.

Finally, how the eating disorder phenomenon can be addressed regarding healthcare is an essential factor in preventing EDs. It is relevant that adolescents receive information about the health risks associated with these disorders and learn to recognize their signs and symptoms to seek appropriate help. In the same way, it is essential to promote healthy eating habits, strengthen self-esteem, address risk factors, and provide emotional and social support, not only from home, but in familiar environments such as sports practice.

Prevention in schools and health institutions is necessary. They should consider the children and adolescent population as the focus of the programs, which must be practical, multidimensional, and based on empowering the participation of children and adolescents, and their families, relatives, coaches, and peers. Without any doubt, public policies are necessary that can impact the entire national territory.

These results allow us to point out that, to reduce the incidence of eating disorders in adolescent athletes, it is necessary, as soon as possible, to assess the relationship between the years of sports practice and the type of sport. It is also paramount to implement prevention programs for teaching to broaden personal goals and perspectives, break social stereotypes, be aware of the importance of hours of rest and sleep, healthy eating, respect for oneself and others, and resistance to frustration, among others. In addition, it is mandatory to limit exposure to media linked to body image issues until athletes have a realistic, objective, and healthy image of themselves. Professional counseling is essential as it will allow the athlete to know themself better, accept their uniqueness, and learn to control the manipulation of the environment.

## 6. Limitations and Implications of the Study

Among the limitations of this study, we highlight the lack of some variables that may have influenced the aim of this study, such as environmental factors, recall biases, context pressure, and self-esteem. Likewise, adolescence was considered as the period between 10 and 19 years of age, according to the UNICEF definition. This is a limitation due to the difference that exists in these ages. Also, as the EAT-26 was self-applied, there could have some misunderstanding and answer biases.

Based on the observed limitations, we recommend dividing the population into preadolescent and adolescent groups for further analysis. Ensure that the participants are free of pressure when answering the questions and know about the athletes’ ideas about eating disorders before applying the questionnaire. For future studies and to increase the validity of their responses, it is recommended to implement face-to-face interviews, as well as the presence of a psychologist to safeguard the welfare of the participants and avoid biases. Finally, a relevant recommendation is to conduct specific research based on the risk of ED depending on the characteristics of each type of sport and research deeply into those that are high aesthetic implications.

## Figures and Tables

**Table 1:** Sociodemographic data of participants.

ED Risk	Frequency	Percentage
No Risk	279	78.8
At risk	75	21.2
Total	354	100.0

Legend: ED = eating disorders.

**Table 2:** Results of the mean differences between the risk variables of eating disorders with variables of sex, housing area, and sports profile.

Variable	Category	N	$\bar{x}$ (SD)	t	Sig. (Bilateral)	ES	95% CI	
							Lower	Upper
Sex	Woman	172	13.19 (12.50)	0.155	0.877	0.197	−2.308	2.702
	Man	182	12.99 (11.46)					
Housing area	Urban	287	12.83 (11.84)	0.838	0.402	1.361	−1.832	4.554
	Rural	67	14.19 (12.47)					
Last competition results	Podium	228	12.65 (12.10)	0.924	0.356	1.227	−1.384	3.839
	No podium	126	13.88 (11.70)					
Level of competition	National	322	12.41 (10.94)	−3.44	0.001	−7.52	−11.82	−3.233
	International	32	19.94 (18.34)					
Sport sector	Conventional	328	13.05 (11.60)	−0.23	0.817	−0.56	−5.366	4.233
	Paralympic	26	13.62 (16.07)					
Type of Sport	Individual	240	12.15 (11.13)		0.032	−2.907	−5.569	−0.245
	Collective	114	15.06 (13.37)	−2.148				

Legend: N = sample; SD = standard deviation; ES = effect size; CI = confidence interval.

**Table 3:** Results of the mean differences between the variable risk of eating disorders with educational variables, socioeconomic stratum, and means of transportation.

Total ED		$\bar{x}$ (SD)	F	Sig.
Grouped age	Preadolescent	9.79 (10.57)	1.395	0.249
	Adolescent	13.49 (12.03)		
	Young adult	11.09 (12.12)		
Institution	Do not study	13.20 (12.74)	0.678	0.508
	Private	11.76 (10.49)		
	Public	13.55 (12.31)		
Level of Institution	Primary	6.64 (7.63)	2.561	0.079
	Secondary	13.56 (12.03)		
	Technology	11.60 (12.08)		
Socioeconomic stratum	1	14.11 (12.93)	1.111	0.351
	2	13.04 (11.10)		
	3	11.35 (11.00)		
	4	9.25 (7.01)		
	5	13.81 (14.54		
Media of transportation	Car	11.66 (12.65)	1.237	0.295
	Bicycle	10.19 (7.71)		
	Bus	13.55 (11.38)		
	Motorcycle	12.58 (11.62)		
	Walking	14.48 (12.87)		

Legend: SD = standard deviation; Sig. = significance.

**Table 4:** Correlations of risk of eating disorder with the sports profile.

		Age	Last Competition Result	Hours of Training per Day	Number of Trainings per Day	Training Days a Week	Years in the Sport
Total Eating Disorders	r	0.017	−0.032	0.136 *	0.146 **	0.045	0.153 **
	Sig.	0.748	0.545	0.011	0.006	0.402	0.004

** The correlation is significant at level 0.01 (bilateral). * The correlation is significant at level 0.05 (bilateral).

**Table 5:** Explanatory model of the risk of eating disorder.

	Expected sign	Model Coefficient	β	t	Sig.	FIV
(Constant)		21.166		5.87	0.000 **	
Body appreciation	+	−0.347	−0.245	−4.822	0.000 **	1.026
Years in the sport	+	0.769	0.163	2.882	0.004 **	1.263
Individual and collective	+	1.416	0.055	0.988	0.324	1.247
Type of last competition	+	6.836	0.164	3.249	0.001 **	1.011
R^2^		0.12				
Adjusted R^2^		0.109				
Durbin−Watson		1.828				
N		354				

Legend: β = Beta coefficient; Sig. = Significance; FIV = Variance Inflation Factor. Note: Dependent variable: Total ATT risk. ** *p* < 0.10, 0.05.

## Data Availability

The data presented in this study are available on request from the corresponding author.
